# Development and evaluation of a virtual patient-centered outcomes research training program for the cystic fibrosis community

**DOI:** 10.1186/s40900-021-00328-4

**Published:** 2021-12-04

**Authors:** Emily M. Godfrey, Erin K. Thayer, Laura Mentch, Traci M. Kazmerski, Georgia Brown, Molly Pam, Morhaf Al Achkar

**Affiliations:** 1 Department of Family Medicine, School of Medicine, University of Washington, 4311 11th Ave NE, Box 354982, Seattle, WA 98105 USA; 2 Cystic Fibrosis Reproductive and Sexual Health Collaborative, Seattle, USA; 3 Department of Pediatrics, School of Medicine, University of Pittsburgh, Pittsburgh, PA USA

**Keywords:** Cystic fibrosis, Co-development, Education, Evaluation, Patient-centered outcomes research, Patient involvement, Patient engagement, Training

## Abstract

**Background:**

Patient-centered outcomes research (PCOR) emphasizes patient-generated research priorities and outcomes, and engages patients throughout every stage of the research process. In the cystic fibrosis (CF) community, patients frequently provide input into research studies, but rarely are integrated onto research teams. Therefore, we developed and evaluated a virtual pilot PCOR training program to build PCOR capacity in the CF community (patients, caregivers, researchers, nonprofit stakeholders and providers). We aimed to show changes among participants’ perceived PCOR knowledge (a.k.a PCOR knowledge), confidence in engaging stakeholders, and post-training session satisfaction.

**Methods:**

Guided by a prior CF community educational needs assessment, our researcher and patient-partner team co-developed a four-part virtual online training program. We structured the program towards two learner groups: patients/caregivers and researchers/providers. We evaluated participants’ PCOR knowledge, confidence in engaging stakeholders, and session satisfaction by administering 5-point Likert participant surveys. We tested for significant differences between median ratings pre- and post-training.

**Results:**

A total of 28 patients/caregivers, and 31 researchers/providers participated. For both learner groups, we found the training resulted in significantly higher PCOR knowledge scores regarding “levels of engagement” (p = .008). For the patient/caregiver group, training significantly increased their PCOR knowledge about the barriers/enablers to doing PCOR (p = .017), effective PCOR team elements (p = .039), active participation (p = .012), and identifying solutions for successful PCOR teams (p = .021). For the researcher/healthcare provider group, training significantly increased participants’ ability to describe PCOR core principles (p = .016), identify patient-partners (p = .039), formulate research from patient-driven priorities (p = .039), and describe engagement in research grants (p = .006). No learner group had significant changes in their confidence score. Most participants were either “satisfied” or “very satisfied” with the training program.

**Conclusions:**

Overall, our virtual pilot PCOR training program was well received by patients, caregivers, researchers and providers in the CF community. Participants significantly improved their perceived knowledge with core PCOR learning items.

*Trial registration* Retrospectively registered at clinicaltrials.gov (NCT04999865).

## Plain English Summary

Cystic fibrosis (CF) is a rare, genetic disease; meaning people are born with the disease and have it throughout their lives. CF is a multi-system disease, primarily affecting the respiratory system. Daily care for people with CF (PwCF) includes taking many medications and breathing treatments. Many PwCF have experienced participating in research as research subjects, but not as partners on research teams participating as experts with the lived experience. Including patient and caregiver partners on research teams is important to improve quality of research. By working alongside each other, patient and/or caregiver partners and researchers build trust. Together, they pursue research questions deemed most important to patients and through this inclusive process, study findings are more rapidly adapted by patients and their communities. We found in a prior survey that 85% of patients, caregivers, healthcare providers and researchers in the CF community wanted training on how to include patient partners onto research teams. Our researcher and patient-partner team co-developed a four-part virtual pilot online training program focused on how to integrate patients/caregivers into research teams. This study aims to show changes in participants’ perceived knowledge, confidence in engaging stakeholders and satisfaction after participating in our training program. Participants included patients, caregivers, researchers and health care providers. We found that our training program improved knowledge about patient-engaged principles for all participants. This training program stands ready to serve as a model for further development to help increase capacity around patient and stakeholder engagement on research teams in the CF community.

## Background

More than 30,000 people in the United States have cystic fibrosis (CF), which is a rare, life-shortening, multi-organ disease that can lead to severe respiratory and digestive problems as well as other complications such as infections and diabetes [1]. Until fairly recently, most persons affected by CF were children, but today, with increased medical interventions, more than 50% of people with CF (PwCF) are adults with a median survival of almost 45 years [1]. The CF community is widely recognized for its long-standing tradition of including PwCF and families to help shape research affecting their community [2, 3]. However, this patient participation has been limited to only discrete parts of the research process, such as participating on data safety monitoring boards, prioritizing research topic areas, providing feedback on study questionnaires or reviewing grant proposals. While this level of involvement, according to the spectrum of patient/stakeholder engagement, allows patients to provide input, it falls short of genuine engagement and partnership with researchers [4]. Part of what makes bringing PwCF together onto research teams so difficult are strict infection control guidelines that restrict in-person contact between patients to avoid the spread of deadly pathogens [5]. This is especially problematic for traditional methods of patient engagement, which are mostly geared for “in-person” group interactions.

Patient-centered outcomes research (PCOR), or patient and public involvement (PPI), entails meaningfully engaging patients, caregivers and other stakeholders (such as clinicians, payers and policy makers) throughout the research process and is increasingly gaining traction among research teams in the United States [6]. Patients, in particular, are valuable to include on research teams because they provide expertise in living daily with their disease. Ideally, research teams using PCOR methodology invite patients as partners to bring ideas and questions based on their lived experience, with researchers then sharing a variety of possible approaches to study them. With this exchange, patient-partners begin to understand the research process more fully and can move towards authentically participating in all phases of research. PCOR has shown to improve research quality, increase patient trust in both the research and researchers, and positively affect health outcomes [6–8].

The process of inviting patients as full partners onto research teams requires a cultural shift among researchers who prize efficiencies, and rarely have to contend with diverse perspectives, new unconventional possibilities, or members of the team who are unfamiliar with research terms and processes [9]. For researchers and patients who want to learn about PCOR, training is available. Current PCOR training curricula, however, do not address certain cultural aspects unique to the CF community. For example, power dynamics exist in all areas of medicine, but the hierarchical patient-doctor relationship in CF is considered to be especially apparent because of the life-long and complex nature of the disease [10, 11]. Clinicians are unsure how to ask patients to serve as partners without patients feeling a sense of obligation [12]. Additionally, as a rare disease, PwCF, caregivers, clinicians and researchers tend to already know one-another, and thus the change from role as patient to patient-partner is more difficult. For example, when we first started our PCOR team, patient partners were reluctant to speak freely in front of researchers and clinicians due to the fear that something they would say would get back to their personal clinician [13]. This made patients want to keep their discussions confidential. Thus, based on our prior needs assessment, we found the most important training areas to address for both the patient/caregiver and provider/researcher groups included: (1) knowing the time commitment required to learn PCOR methodology, and (2) learning how to develop and maintain trust when patients/caregivers are active members of the research team [14]. PCOR training is necessary to achieve a critical mass of researchers employing this methodology. Additionally, more research funders require stakeholder engagement on grant submissions. This study aims to evaluate a virtual training adapted for the CF community on perceived PCOR knowledge acquisition, confidence in engaging stakeholders and satisfaction of the training program.

## Methods

We report this study according to the GRIPP2 guidelines in the reporting of patient and public involvement (PPI) in research [15]. We report as many elements on the checklist as relevant to this study.

### Design

**Fig. 1:**
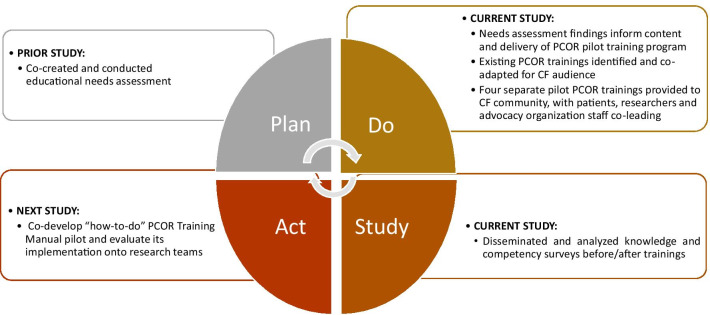
Adapted Model For Improvement framework to build patient-centered outcomes research capacity in the cystic fibrosis community

We employed a qualitative descriptive design to co-develop four training sessions with patient and advocacy organization stakeholders using a framework adapted from the Model for Improvement [16]. The Model for Improvement framework uses four iterative phases (1) Plan, (2) Do, (3) Study, (4) Act (see Fig. 1) [16]. Our focus for this present study were phases (2) and (3). Phase 1 (Plan) included a needs assessment co-produced and conducted by our team, which has been published previously [14].

### Setting

This study was conducted virtually using Zoom for synchronous meetings and training sessions, Slack and email for asynchronous communications between team members, and Google Docs for document sharing. The origins of this co-production patient-engaged team came from the Cystic Fibrosis Reproductive and Sexual Health Collaborative (CFReSHC, cfreshc.org). CFReSHC is a U.S-based, nationwide, English-speaking, online patient-driven collaborative committed to responding to research gaps articulated by adult PwCF. In 2018, having built a successful patient-engagement structure, members of CFReSHC wanted to build PCOR capacity within the greater CF community. CFReSHC researcher and patient members co-wrote a successfully funded grant application to the Patient-Centered Outcomes Research Institute (PCORI) to conduct a needs assessment and develop and evaluate an educational PCOR training program for PwCF, researchers and health care providers. CFReSHC is hosted by the University of Washington Department of Family Medicine.

### Training co-developers: participant characteristics

The training development and evaluation team consisted of several different types of stakeholders, including clinician-researchers (n = 3), PwCF (n = 3), public health researcher (n = 1) and a CF advocacy organization staff member (n = 1). The project team was led by a CFReSHC co-founder and a practicing family physician and clinician-researcher with patient engagement methodological expertise. A second clinician-researcher specialized in CF, and the third clinician-researcher was an expert in educational design and evaluation. Project co-leads included three current CFReSHC patient-partners: (1) one with a career as a sexual health educator and trainer, (2) another with CF community advocacy connections, and (3) another with experience in media and marketing. Our community advocate was a staff member for the largest non-for-profit organization in the CF community in the United States, the Cystic Fibrosis Foundation (CFF). A Master-level student in the University of Washington School of Public Health managed the team and performed the data analysis.

### Training program development

**Fig. 2:**
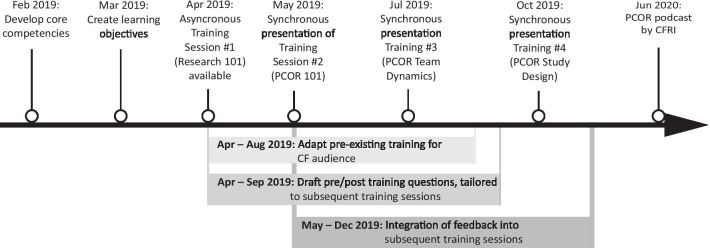
PCOR training development activities Feb 2019-Jul 2020

We developed, led and evaluated four separate pilot trainings between February 2019 and August 2020. Members of the development/evaluation team met weekly throughout the study period. The team’s activities are depicted in the timeline in Fig. 2, which were guided by best practices for training and development provided by the University of Washington Institute of Translational Health Sciences [17]. Our initial steps included developing four core competencies and learning objectives for each competency, informed by findings from a prior CF community educational needs assessment [14]. Over the course of several weeks, the team co-created four separate core competencies for two distinct learner groups: (1) researchers/providers and (2) patients/caregivers. We iteratively developed 3–6 learning objectives within each core competency using Bloom's taxonomy until consensus was reached by all team members [18]. To create the training format, we applied the key adult learning principles, which included pre-training materials, learning aids, and multi-modal learning strategies (e.g., didactics, small group activities, case scenario discussions, and question/answer sessions) [19, 20]. The public health researcher and a patient-partner with sex-education experience then collaboratively scouted the internet for existing PCOR training programs with materials or items that met our learning objectives (see Appendix 1). The patient-partner with CF advocacy organizations connections sought pre-existing training programs already available for the CF community. At each weekly meeting, the public health researcher and patient-partners would present power-point slides or material they found on the internet, and the remaining team members adapted these to meet items specifically mentioned during our needs assessment [14]. The public health researcher and two patient-partners additionally identified information from the peer-reviewed literature on PCOR, which supplemented aspects of the CF-specific training program that had not been identified in pre-existing PCOR training [21–29]. The literature was reviewed by the clinician-researchers and the educational specialist. Because in-person contact between patients with CF is restricted, we included information about the use of web-based platforms for virtual PCOR collaborations based on an in-depth interview study performed by our team [30].

### Data collection

Once the materials to conduct a training session were finalized, we co-led the online PCOR training program with participants from the CF community throughout the United States. We invited adults with CF and their caregivers, CF providers, researchers and research staff to participate in and provide feedback about our training sessions. We advertised the training through CFReSHC, CFF, Cystic Fibrosis Research Institute (CFRI), and the University of Washington. The patient-partner with marketing skills created recruitment flyers for each training and widely advertised the training throughout the CF community. Participants who attended a training session and completed the surveys were provided a $15 gift card.

All team members helped conduct four separate pilot PCOR training sessions between April 2019 and October 2019. We evaluated each training session by administering a survey to participants before and immediately after the training session, except for Training 1, where we surveyed participants only after the training. The survey questions asked participants to rate their agreement about their own perceived PCOR knowledge (a.k.a PCOR knowledge), confidence with engaging stakeholders and training session satisfaction (post-training only) using a 5-point Likert scale. At the end of each survey, we asked open-ended questions regarding what the participants liked about the training session and how we could improve. After each training session, the development team met for 45 min to discuss what went well and what could be improved, which were captured as notes to implement into the next iteration of the training program. A single, summative PCOR session for the CF community was provided by the researcher–clinician co-lead and a patient-partner as a podcast through CFRI in July, 2020 [31]. This podcast was not evaluated by our team.

### Data analysis

The public health researcher performed the statistical analysis of the survey responses and created the data tables and figures. We conducted descriptive statistics for participant characteristics and median scores with inter quartile range (IQR) for post Training 1 (Research 101). For Trainings 2–4, we calculated the median pre- and post-training score for each PCOR knowledge and confidence in engagement questions. We also assessed the difference between training attendees’ self-rated pre- and post-training responses using the sign test. Because the sample size was small and not normally distributed, we used non-parametric summary statistics and tests. We used the sign test specifically to assess whether there was a significant directional change in the pre- and post-training responses for individual responses (alpha = 0.05) [32]. We performed statistical analysis using R version 3.6.3 with RStudio version 1.3.1093 [33]. We summarized responses to open-ended questions after each training session regarding suggestions for improvement. The patient-partners provided input on how best to display the results with tables and figures.

## Results

### Training program content

Our pilot PCOR training program consisted of four separate core competencies: (1) Understand the principles of research; (2) Understand the science of PCOR; (3) Participate in and maintain a PCOR team; (4) Design and implement a PCOR study. Each core competency served as a separate session title and within each competency we listed learning objectives, training format, presenters/facilitators and the learner group/audience for which the session was intended (see Appendix 2). Our first training was intended for patients/caregivers only (Research 101), and included a 25-min asynchronous, self-directed learning seminar intended to be viewed before the subsequent interactive PCOR sessions. The remaining three training programs were synchronous, interactive training sessions, lasting approximately 1.5 h each. Two of these sessions included both learner groups (patients/caregivers and researchers/providers) together (PCOR 101 and PCOR Team Dynamics) and one session (PCOR Study Design) was for researchers/healthcare providers only. The pilot training sessions can be downloaded here: familymedicine.uw.edu/pcor-guide/.

### Training program evaluation

Training program participants included 28 patients and caregivers, and 31 researchers and providers. Several participants attended more than a single PCOR session. Detailed training program participant characteristics, including type of participant, job title, and attendees per session are reported in Table 1.

**Table 1:** Training program participant characteristics

	N (%)
Total unique participants* (n = 59)	
Patients/caregivers	28 (48)
Researchers/providers	31 (53)
Clinic staff	3 (10)
Nurse	3 (10)
Physician/advanced practice provider	8 (26)
Researcher	8 (26)
Social worker	3 (10)
CF community organization	4 (13)
Student	1 (3)
Missing	1 (3)
*Training 1: Research 101 (n = 17)*	
Patients/caregivers	17 (100)
Providers/researchers	N/A
*Training 2: PCOR 101 (n = 26)*	
Patients/caregivers	15 (58)
Providers/researchers	11 (42)
*Training 3: PCOR team dynamics (n = 20)*	
Patients/caregivers	15 (75)
Providers/researchers	5 (25)
*Training 4: PCOR Study Design (n = 21)*	
Patients/caregivers	N/A
Providers/researchers	21 (100)

^*^Some participants attended more than one training session

Overall, participants significantly improved self-assessed PCOR knowledge. The median Likert scale responses post-training for training session 1 and a test of the difference between knowledge perception questions administered before and after training sessions 2, 3, and 4 are presented in Table 2.

**Table 2:** Participant self-assessment of PCOR knowledge Likert scale responses pre- and post-training

Training 1: research 101 (patients/caregivers only)	Patient/caregiver (n = 17)					
	Median response [IQR]					
	Post only					
I can describe the different types of research methods used in PCOR	4 [4, 4]					
I can describe the processes, sections, and terminology of a research grant	4 [4, 4]					
I can describe the processes of disseminating study findings (e.g., publication, poster, oral presentation)	4 [4, 5]					

Training 2: PCOR 101 (both learner groups)	Patient/caregiver (n = 15)			Researchers/providers (n = 11)		
	Median response		Difference	Median response		Difference
	Pre	Post	p value	Pre	Post	p value
I can identify the benefits and value of patient/caregiver engagement in research	4	5	0.13	4	5	0.13
I can define the levels of patient engagement, from minimal to control	3	4	**0.008**	3.5	5	**0.031**
I can describe and provide examples of the core principles of PCOR	3	4	0.11	3	5	**0.016**
I can articulate how PCOR findings improve health in the community, raise awareness, and increase patient advocacy	4	4	1	4	5	0.063
I can identify barriers to adopting PCOR and enablers to undertaking this type of research	4	4	**0.017**	4	5	0.13
I can identify ways to turn PCOR work into academic productivity **(HCP only)**	NA	NA	NA	4	4	0.063

Training 3: PCOR team dynamics (both learner groups)	Patient/caregiver (n = 15)			Researchers/providers (n = 5)		
	Median response		Difference	Median response		Difference
	Pre	Post	p value	Pre	Post	p value
I can describe the elements of an effective PCOR team	3	4	**0.039**	2	5	0.25
I can describe how to create conditions for patient/caregiver partners to be active participants within a PCOR team at every step of the research process **(HCP only)**	NA	NA	NA	2	5	0.063
I can describe how to be an active participant in a PCOR team in every step of the process **(pts only)**	2	4	**0.012**	NA	NA	NA
I can identify barriers to successfully functioning PCOR teams	4	4	**0.004**	2	5	0.13
I can articulate potential solutions to address barriers to successfully functioning PCOR teams	4	5	**0.021**	3	4	0.063
I know how confidentiality of patient/caregiver partners will be maintained on a PCOR team	4	5	0.18	4	5	0.25

Training 4: PCOR study design (researchers/providers only)	Researchers/providers (n = 21)					
	Median response		Diff			
	Pre	Post				
I know how to identify patients and caregivers to participate as partners in research	4	4	**0.039**			
I know how to formulate research questions from patient-driven priorities	4	4	**0.039**			
I can describe successful components of patient engaged research in a grant application	4	4	**0.006**			
I can articulate the role of patient and caregiver partners at every stage of the research project	3	4	**0.001**			

P values noting significant differences between pre- and post training are bolded. (1 = strongly disagree, 2 = disagree, 3 = neutral, 4 = agree, 5 = strongly agree)

After Training 1 (Research 101), we found the majority of patients/caregivers reported being able to describe the different types of research methods (15/17, 88%), terminology (14/17, 82%), and modes of dissemination used in PCOR (17/17, 100%).

After Training 2 (PCOR 101), we found a significant difference in change of PCOR knowledge related to engagement levels compared to before among patients/caregivers and CF researchers/providers, (p values = 0.008 and 0.031, respectively). Following the session, patients/caregivers reported being significantly better able to identify barriers and enablers to adopting PCOR compared to pre-training (p = 0.016), whereas CF researchers/providers reported being significantly better able to describe and provide examples of the core principles of PCOR (p = 0.017).

During Training 3 (PCOR Team Dynamics), patients/caregivers significantly improved their PCOR knowledge in every aspect of the training except knowing how confidentiality of patient partners are maintained on PCOR teams. In contrast, we found no reported significant PCOR knowledge changes among CF researchers/providers. After this session, patients/caregivers reported being significantly better able to describe elements of an effective PCOR team (p = 0.039), how to be an active participant throughout the research process (p = 0.012), how to identify barriers to successfully functioning PCOR teams (p = 0.021).

In Training 4 (PCOR Study Design for CF Researchers/Providers only), participants reported significant improvement of their PCOR knowledge in every aspect of the training, including how to identify patients and caregivers to participate as partners in research, formulate research questions from patient-driven priorities, articulate successful components of an engagement plan in grant applications, and describe the patient partner role at every stage of the research project (p-values = 0.039, 0.039, 0.006, 0.001).

#### Confidence with engaging partners in PCOR

Confidence was only solicited in surveys related to Training 2–4. The median confidence score of patient/caregiver participants attending training sessions 2 (PCOR 101) or 3 (PCOR Team Dynamics) to engage as a partner in research was a “4” (fairly confident), which did not change significantly after either training. Similarly, the median confidence score of CF researchers/providers attending training sessions 2, 3 or 4 (PCOR Study Design) did not change significantly: self-rated confidence to engage PwCF in research before each training was either “3” (neutral) or “4” (fairly confident), and after each training was “4” (fairly confident).

#### Training session satisfaction

**Fig. 3:**
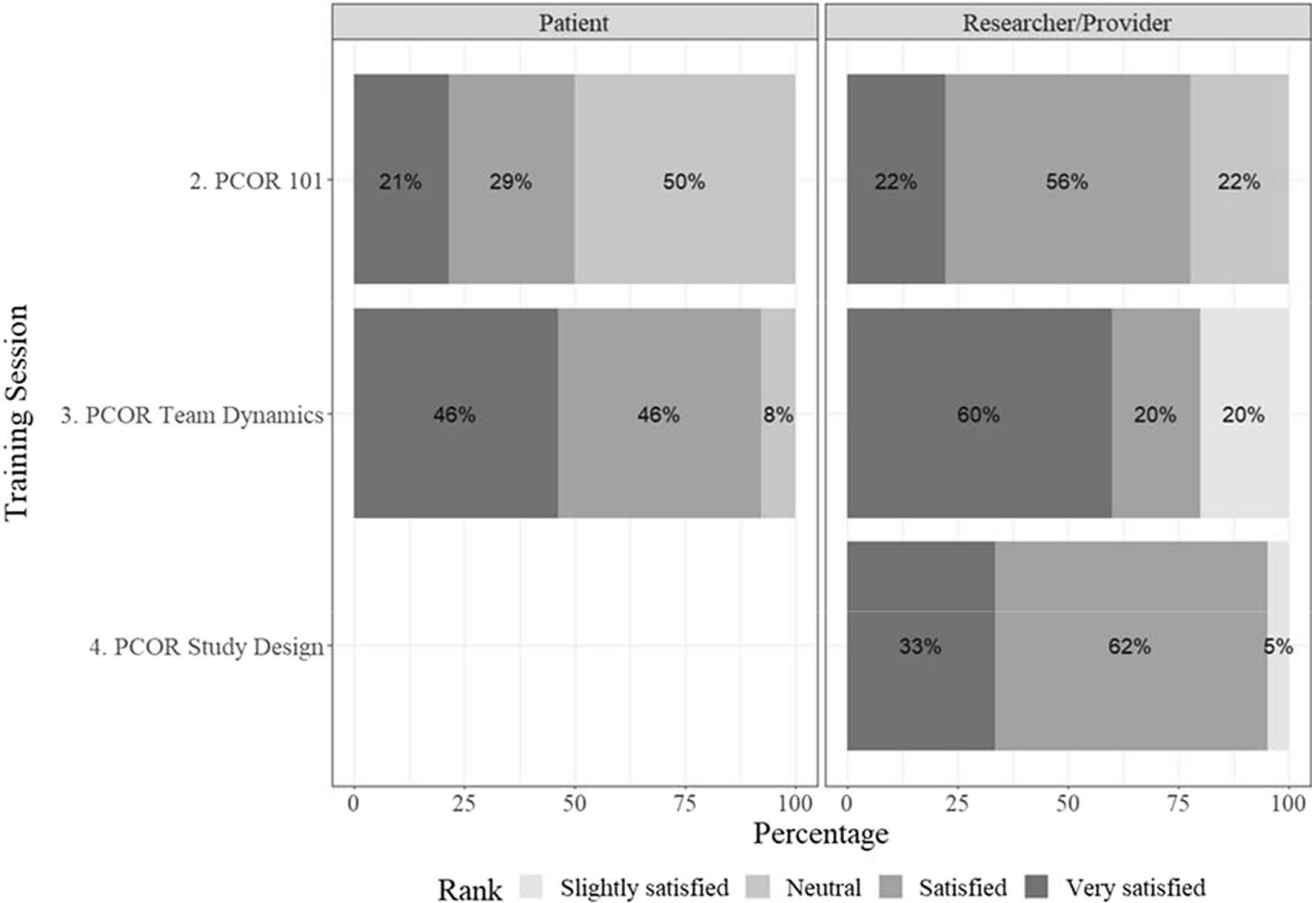
Satisfaction with the interactive training session format, by participant type

Overall, both learner groups were satisfied with the format of each training session (Fig. 3). The highest proportion of participants from either the patient or researcher/provider group who reported being very satisfied with the training occurred with Training 3 (PCOR team dynamics) compared with the other training sessions.

#### Training satisfaction: open-ended questions

*Training 1 (Research 101)* The most common beneficial aspects participants noted was learning about research terminology, the grant submission process, how to design research questions, and the difference between types of research studies (e.g., quantitative vs qualitative and retrospective vs prospective). Most participants appreciated the inclusion of multiple speakers, including people with CF and the incorporation of visuals.

*Training 2 (PCOR 101)* Most participants liked the interaction between patients, caregivers, researchers and healthcare providers and the fact that the learners were taught together. Some participants reported not liking required participation in the interactive portions of the training, while others thought the breakout sessions were too small and too short. One person suggested having facilitators participate in each group to help move the conversation along.

*Training 3 (PCOR Team Dynamics)* This training included two case scenarios related to: (1) creating a respectful space for collective sharing, and (2) building and maintaining trust. Within each scenario, participants were asked to identify barriers to a successfully functioning PCOR team and articulate potential solutions to address those barriers. All participants commented that they enjoyed the discussion of both case scenarios, and thought they clearly illustrated potential challenges PCOR teams face. Participants also liked how facilitators engaged to advance the discussions. For example, one researcher/provider participant noted that all participants were “encouraged to speak”. Participants suggested including more role playing for this training.

*Training 4 (PCOR Study Design)* Participants favorably rated the video conferencing format and thought the session was easy to join. Several suggestions for improvement included: Offering closed captioning versions of the presentation for viewing in different languages (including English for Deaf viewers); shorten the panel discussion and bring back the interactive breakout session format; provide more specific examples of how including patients or caregivers on the research team improves the quality and relevance of research; include example documents, such as a patient partner biographies and a list of potential patient partner roles; include more discussion of ways to include a diverse representation of patients on the research team.

## Discussion

In this study, our team, consisting of researchers, clinicians, PwCF and CF advocates, developed and evaluated four unique virtual training sessions related to patient/caregiver engagement on CF research teams. Based on the results of our prior educational needs assessment [14], we worked with an educational specialist to identify competencies and used an iterative process to specify our learning objectives based on the Model of Improvement [16]. We customized existing PCOR materials to meet the needs of the CF community, which called for incorporation of key adult learning principles, including pre-training materials, learning aids, and multi-modal learning strategies (e.g., didactics, small group activities, discussions, and question/answer sessions). Our findings suggest participants improved their knowledge about PCOR after each training session. Participants had fairly high confidence about their own PCOR skills at baseline, a measurement which did not significantly change with the PCOR training. Regardless of change in PCOR knowledge or confidence, participants in both learner groups (researchers/providers and patients/caregivers) were very satisfied with the teaching sessions. With high confidence and satisfaction, it is likely that learners who attended the sessions felt favorably about PCOR going into the sessions, but the change in baseline knowledge suggests that this filled a learning gap for the CF community.

This study is unique because of the level of engagement of our patient- and stakeholder-partners who maintained input into the project starting at the grant generation stage through dissemination of the findings. Our process of highlighting patient-partner skills (in addition to their insights as persons with the disease) allowed our patient-partners to more fully contribute to team activities. Additionally, open-ended comments from training participants indicated positive feedback in having PwCF and researchers teaching together. The input of the patient-partners helped make the didactic material more relatable to the audience. The impact of PPI in this work enabled our team’s patient-partners to intimately learn about PCOR. As a result, they wrote and published an article about PCOR in a widely read quarterly periodical by CF patients [34] and produced an asynchronous podcast for the CF community that is publicly available [31].

As major funding agencies increasingly encourage and expect the inclusion of patient stakeholders on grant applications (i.e., by making such engagement a requirement for funding) [35], patient and caregiver partners will increasingly begin to join CF clinical research teams and accordingly, CF researchers will need adequate skills to successfully integrate these members. Engaging patients and caregivers for the long-term requires a sustained approach to support CF researchers and team collaborations, and to ensure PCOR principles of belonging and collaborative learning are actualized [20]. Other PCOR training programs such as one developed by the National Organization of Rare Disorders (NORD) in conjunction with the University of Maryland also contains the notion of sustaining support for PCOR by developing a pipeline of qualified and skilled mentors in PCOR methodology for new PCOR teams [23]. A separate study found that training priorities should include helping team members identify appropriate patient partners, devising an engagement strategy that clarifies roles and expectations, and building skills for positive team dynamics [36]. Prior training suggests that learning is not a singular one-time event, but comes from the act of “doing.” Thus, ideally research teams should have an opportunity to participate in a PCOR mentoring program in which they can integrate patient/caregiver partners and have experts with whom to consult.

Our study had some limitations. Per the GRIPP2 guidelines, we did not quantitatively measure the impact of PPI in this study. We did, however, collect qualitative feedback about the impact of PPI from training participants, many of whom indicated positive comments about seeing PwCF as leaders of this work. Another limitation of this study was the low participation rates, which we believe was primarily due to our recruitment strategy. We had intended for the program to be presented sequentially with the same participants attending each training session. Thus, we initially limited our invitations to only those participants who had attended the prior session. We eventually opened our invitations to CF community members who had participated in our prior needs assessment, but finding a mutual time for synchronous training was difficult to achieve with busy work schedules. Our program evaluation was limited to feedback occurring immediately post-training. We did not include an evaluation process that assessed whether attendees later incorporated PCOR onto their research teams, or whether patients/caregivers joined research teams after receiving the training.

## Conclusions

A core team of researchers, patient-partners and advocacy stakeholders successfully co-developed four pilot PCOR training programs and a publicly available podcast about how to increase patient-engagement capacity on research teams. This CF-specific PCOR training was well received by patients, caregivers, health care providers and CF researchers. The program significantly improved PCOR knowledge with core PCOR learning items. The training development team is now creating a new, comprehensive PCOR training manual with input from stakeholders from the CF community, with the goal of increasing interest in PCOR skills and methods among CF clinical researchers.

## Data Availability

The authors confirm that trainings, data collection tools are available within the article [and/or] supplementary materials. The datasets for the current study are not publicly available, but will be made available from the corresponding author on reasonable request.

## Appendix 1: PCOR training—adapted materials

Our team conducted an online search for existing PCOR training materials. Identified materials were mapped to specific competencies and adapted for our specific training goals, core competencies and learning objectives. A summary of the materials adapted for use in our training is provided below.

**Table:**

Teaching concept	Training theme	Pre-existing resource identified
**Why engage patients and caregivers in research?**		
*Better research through engagement*	How engagement helps us do our work Why engagement matters Patient and stakeholder involvement Strengthening the PCOR community Advancing engagement & influencing others	https://www.pcori.org/sites/default/files/PCORI-Better-Research-Through-Engagement.pdf
*Research done differently*	What is PCOR What do we mean by patient-centered PCORI funding for patient-centered studies Testimonials	https://www.pcori.org/sites/default/files/PCORI-Research-Done-Differently.pdf
*Impact of engagement in research*	Evaluation framework for assessing short- and long-term impact of engagement What PCORI considers engagement Effect of engagement on study design, processes, and outcomes selection	Forsythe, L., Heckert, A., Margolis, M.K. et al. Qual Life Res (2018) 27: 17. https://doi.org/10.1007/s11136-017-1581-x
**How to engage patients and caregivers as partners in research**		
*Research fundamentals*	Comprehensive training package to learn about the research process and to be involved in PCOR	https://www.pcori.org/engagement/research-fundamentals
*Initiating partnerships for PCOR*	How researchers can engage patients and stakeholders to improve patient-centered research Where to find potential research partners Lessons learned about initiating research partnerships	https://www.pcori.org/sites/default/files/PCORI-Engagement-Strategies-for-Initiating-Research-Partnerships-Info-Sheet-71917.pdf
*Developing research partnerships*	Forming partnerships with patients and other stakeholders Where and how to find partners Considerations in clinician partnerships Lessons learned from PCORI funded research teams	Anyanwu C, Hemphill R. Finding and Recruiting Research Partners: Lessons from PCORI Awardees. PCORI Engagement Blogs. Sep 1, 2017. https://www.pcori.org/blog/finding-and-recruiting-research-partners-lessons-pcori-awardees
*True partner engagement*	Defining partnerships and engagement in research Principles of authentic partnerships Strengths and weaknesses of full patient engagement in research Time and resources needed for patient-engaged research How to evaluate partnerships	http://trailhead.institute/wp-content/uploads/2017/04/truepatientpartnerengagement_final.pdf
*Roles of patient and caregiver partners in research*	Common patient-partner activities and effects of partner engagement Examples of effects of partner engagement in planning, conducting, and disseminating phases of a study	https://www.pcori.org/sites/default/files/PCORI-Engagement-in-Research-Making-a-Difference-Webinar-Info-Sheet-091917.pdf
*Initiative to support patient involvement in research (INSPIRE): community workshop report*	Describes how to build infrastructure for patient engagement Discusses mentorship and training to develop meaningful patient engagement and roles for patient-partners in research Discusses different models of engagement (i.e., engagement as part of a research study vs. as part of research institution) Provides recommendations for future patient engagement (e.g., peer-to-peer mentoring program for researchers and patients, research orientation) and for increasing diversity in patient-researcher partnerships	Lavalle DC, Gore JL, Lawrence SO, Lindsay J, Marsh S, Scott MR, Wernli K. Initiative to Support Patient Involvement in Research (INSPIRE): Community Workshop Report [Internet]. October 2016. Available from: https://www.becertain.org/sites/default/files/INSPIRE%20PCOR%20Workshop%20Summary%20FINAL%202016.10.05.pdf
*Stakeholder engagement challenges, strategies, and resources*	Common challenges of partner engagement in research Strategies to prevent or address challenges to engaged research PCORI engagement resources	https://www.pcori.org/sites/default/files/PCORI-Patient-Stakeholder-Engagement-Challenges-Strategies-Resources-Handout-120517.pdf
**How to include PCOR in grant applications**		
*PCORI engagement plan template*	Helps study teams refine engagement plan Guides research team to fulfill the objectives of patient- and stakeholder-engaged research	https://www.pcori.org/sites/default/files/PCORI-Updated-Engagement-Plan-Template.pdf
*PCORI engagement rubric for applicants*	How input from patient and stakeholder partners can be used throughout the research process Provides PCORI engagement principles, definitions, and key considerations for planning, conducting, and disseminating patient-engaged research Provides specific examples of potential partner activities for each part of the PCORI grant application	PCORI Engagement Rubric. PCORI (Patient-Centered Outcomes Research Institute) website https://www.pcori.org/sites/default/files/Engagement-Rubric.pdf Published February 4, 2014. Updated October 12, 2015. Accessed 1/20/2020
*PCORI compensation framework*	Guidelines for compensating patients, caregivers, and organizations engaged in PCORI funded research as research partners Provides varying compensation levels for level of engagement	https://www.pcori.org/sites/default/files/PCORI-Compensation-Framework-for-Engaged-Research-Partners.pdf
*Budget for engagement activities*	Considerations for: Compensation and recognition Patient and caregiver partner expenses Project staff Engagement event costs Incorporating partner feedback	https://www.pcori.org/sites/default/files/PCORI-Budgeting-for-Engagement-Activities.pdf
*PCOR and IRB points to consider*	Planning considerations Issues for IRB submissions and reviews (e.g., standard care vs. research interventions, advertisements, informed consent, HIPAA…)	https://www.partners.org/Assets/Documents/Medical-Research/Clinical-Research/PCOR-and-IRB-Points-to-Consider.pdf
**PCOR 101**		
*Traditional research vs. community-engaged research*	Explains the difference between traditional and community-engaged research and provides examples of both Community-engaged research questions Challenges to community-engaged research and how to overcome these barriers	http://trailhead.institute/wp-content/uploads/2017/04/316043919-community-engaged-research-final.pdf
**PCOR team dynamics**		
*Team science SWOG field guide*	Enabling, reinforcing, and rewarding patient-partner engagement How to support patient-partners in each stage of the research process (design, implementation, dissemination)	https://www.pcori.org/sites/default/files/TeamScience-SWOG-Field-Guide.pdf
*Collaboration and team science*	Characteristics of effective teams Self- and Team-Awareness Understanding team development Building a team Creating a shared vision Sharing recognition and credit Promoting disagreement while containing conflict	Bennett LM, Gadlin H. Collaboration and team science: from theory to practice. J Investig Med. 2012 Jun;60(5):768–75. https://www.ncbi.nlm.nih.gov/pubmed/22525233
*TeamSTEPPS*	Outlines TeamSTEPPS and the phases for delivery A slideset briefing that helps promote TeamSTEPPS to an organization’s leaders and encourage implementation	AHRQ. About TeamSTEPPS. https://www.ahrq.gov/teamstepps/about-teamstepps/index.html AHRQ. TeamSTEPPS 2.0 Leadership Briefing. https://www.ahrq.gov/teamstepps/about-teamstepps/leadershipbriefing.html
**Online collaboration**		
*Overcoming challenges in collaborating online*	Solutions for the following challenges: Communication Establishing and maintaining trust Productivity Lessons learned: Adjust for size of projects Don’t be afraid of social media Play games Train for collaboration Have role clarity but task uncertainty	Challenges to Managing Virtual Teams and How to Overcome Them. Harvard Division of Continuing Education. Blog. https://www.extension.harvard.edu/professional-development/blog/challenges-managing-virtual-teams-and-how-overcome-them Ferrazzi K. How Successful Virtual Teams Collaborate. Harvard Business Review. 2012 Oct. https://hbr.org/2012/10/how-to-collaborate-in-a-virtua
**Evaluating partnerships**		
*Measuring trust in partnerships*	Identifies how trust is conceptualized in health promotion partnerships Provides a 14-item trust measurement tool	Jones J, Barry MM. Developing a scale to measure trust in health promotion partnerships. Health Promotion International. 2011 Feb;26(4):484–491. http://trailhead.institute/wp-content/uploads/2017/04/health_promot__int_-2011-jones-484-91.pdf
**PCOR in the literature**		
*CF example*	Kazmerski T.M., Miller E, Sawicki GS, Thomas P, Prushinskaya O, Nelson E, Hill K, Miller A, Emans SJ. Developing Sexual and Reproductive Health Educational Resources for Young Women with Cystic Fibrosis: A Structured Approach to Stakeholder Engagement. Patient. 2019 Apr;12(2):267–276 https://www.ncbi.nlm.nih.gov/pubmed/30361885	
*Health affairs*	Forsythe LP, Carman KL, Szydlowski V, Fayish L, Davidson L, Hickam DH, Hall C, Bhat G, Neu D, Stewart L, Jalowsky M, Aronson N, Anyanwu CU. Patient Engagement in Research: Early Findings From The Patient-Centered Outcomes Research Institute. *Health Aff.* 2019 Mar;38(3):359–36 https://www.healthaffairs.org/doi/full/10.1377/hlthaff.2018.05067	
*Health expectations*	Shippee ND, Domecq Garces JP, Prutsky Lopez GJ, Wang Z, Elraiyah TA, Nabhan M, Brito JP, Boehmer K, Hasan R, Firwana B, Erwin PJ, Montori VM, Murad MH. Patient and service user engagement in research: a systematic review and synthesized framework. *Health Expect*. 2015 Oct;18(5):1151–66. https://doi.org/10.1111/hex.12090. https://www.ncbi.nlm.nih.gov/pmc/articles/PMC5060820/ This paper uses a systematic review and environmental scan to create an evidence-based framework for patient and services user engagement The framework provides a standard structure and language for reporting and indexing to support comparative effectiveness and optimize PCOR Integral components include: reciprocal relationships, colearning, re-assessment, and feedback The framework describes patient engagement at several stages of research: preparatory, execution, and translational	
*BioMed Central*	Staley K. ‘Is it worth doing?’ Measuring the impact of patient and public involvement in research. BMC Research Involvement and Engagement. 2015;1(6). https://researchinvolvement.biomedcentral.com/articles/10.1186/s40900-015-0008-5 Discusses the current debate around the impact of involving patients/community members in research Provides experiences from researchers engaging in PCOR	
*Implementation science*	Bombard Y, Baker GR, Orlando E, et al. Engaging patients to improve quality of care: a systematic review. Implement Sci. 2018;13: 98. https://link.springer.com/article/10.1186/s13012-018-0784-z Systematic review from 1990 to 2016 for empirical studies that address active participation of patients, caregivers, or families in the design, delivery and evaluation of health services to improve quality of care Identifies strategies and contextual factors that enable engagement of patients in the design, delivery, and evaluation of health services	

## Appendix 2: CF community PCOR training core competencies, learning objectives, training format, presenters and learner groups

**Table:**

At the end of the training program, participants should be able to:			
Learning objectives	Training format	Presenters/facilitators	Learner group(s)/audience
*Competency 1: understand the principles of research (research 101)*			
-To describe the types of research methods used in PCOR -To describe the processes, sections and terminology of a research grant -To describe the processes of disseminating study findings (e.g., publication, poster, oral presentation)	Asynchronous Self-directed learning video Time allotted: 25 min	Didactics presented by two CF researchers and three patient partners with CF	Patients/caregivers
*Competency 2: understand the science of PCOR (PCOR 101)*			
-To identify the benefits and value of patient engagement in research -To define the levels of patient engagement, from minimal engagement to control -To describe and provide examples of the core principles of PCOR -To articulate how PCOR findings improve health in the community, raise awareness, and increase patient advocacy -To understand how to turn PCOR work into academic productivity -To identify barriers to adopting PCOR and enablers to undertaking this type of research	Synchronous Interactive session Time allotted: 90 min Activities: Didactics Small group discussions	Opening comments by a health services research graduate student Didactics by two CF researchers and three patient partners with CF Two 8-min think-pair-share activities (paired discussions) facilitated by two CF researchers and three patient partners with CF	Patients/caregivers Researchers/ healthcare providers
*Competency 3: participate in and maintain a PCOR team (PCOR team dynamics)*			
-To describe elements for successful PCOR team dynamics -To describe how to create conditions for patient-partners to be active participants within a PCOR team at every step of the research process -To identify barriers to successfully functioning PCOR teams and articulate potential solutions to address those barriers -To review strategies to maintain confidentiality of patient-partners when part of the research team	Synchronous Interactive session Time allotted: 90 min Activities: Didactics Small group case-based discussions	Opening comments by a health services research graduate student Didactics by one CF researcher, three patient partners with CF, and a CF stakeholder organization partner 1 15-min small group discussion of two case scenarios facilitated by one CF researcher and three patient partners with CF 1 8-min large group discussion facilitated by one CF researcher and three patient partners with CF	Patients/caregivers Researchers/ healthcare providers
*Competency 4: design and implement a PCOR study (PCOR study design)*			
-To articulate strategies for identifying patient and caregivers to participate as partners in research -To formulate research questions from patient-driven priorities -To identify successful components of patient-engaged research in a research grant application -To articulate the role of patient-partners at every stage of the research project from research question development to the grant writing process to study roll-out	Synchronous Interactive session Time allotted: 90 min Activities: Didactics Short panel presentation Q&A session	Opening comments by a health services research graduate student Didactics by one CF researcher and two patient partners with CF Panel discussion by two guest lecturers (one healthcare provider and one caregiver partner)	Researchers/healthcare providers

## Abbreviations

CF: Cystic fibrosis
CFF: Cystic Fibrosis Foundation
CFReSHC: Cystic Fibrosis Reproductive and Sexual Health Collaborative
CFRI: Cystic Fibrosis Research Inc
IQR: Interquartile range
PCOR: Patient-centered outcomes research
PwCF: People with cystic fibrosis
NORD: National Organization of Rare Disorders
